# Cobalt Oxide Nanograins and Silver Nanoparticles Decorated Fibrous Polyaniline Nanocomposite as Battery-Type Electrode for High Performance Supercapattery

**DOI:** 10.3390/polym12122816

**Published:** 2020-11-27

**Authors:** Javed Iqbal, Arshid Numan, Mohammad Omaish Ansari, Rashida Jafer, Priyanka R. Jagadish, Shahid Bashir, P. M. Z. Hasan, Anwar L. Bilgrami, Sharifah Mohamad, K. Ramesh, S. Ramesh

**Affiliations:** 1 Center of Nanotechnology, King Abdulaziz University, Jeddah 21589, Saudi Arabia; iqbaljavedch@gmail.com (J.I.); phasan@kau.edu.sa (P.M.Z.H.); 2 Department of Chemistry, Faculty of Science, University of Malaya, Kuala Lumpur 50603, Malaysia; sharifahm@um.edu.my; 3 State Key Laboratory of ASIC and System, SIST, Fudan University, Shanghai 200433, China; numan.arshed@gmail.com; 4 Graphene & Advanced 2D Materials Research Group (GAMRG), School of Science and Technology, Sunway University, No. 5, Jalan Universiti, Bandar Sunway, Subang Jaya, Selangor 47500, Malaysia; priyankaj@sunway.edu.my; 5 Department of Physics, Faculty of Science, King Abdulaziz University, Jeddah 21589, Saudi Arabia; rashida.jafer@gmail.com; 6 Centre for Ionics University of Malaya, Department of Physics, Faculty of Science, University of Malaya, Kuala Lumpur 50603, Malaysia; shahidbashirbaig@gmail.com (S.B.); rameshkasi@um.edu.my (K.R.); 7 Deanship of Scientific Research, King Abdulaziz University, Jeddah 21589, Saudi Arabia; alegman@kau.edu.sa

**Keywords:** conducting polymer, polyaniline, cobalt oxide, silver, supercapattery, energy storage device

## Abstract

In this study, silver (Ag) and cobalt oxide (Co_3_O_4_) decorated polyaniline (PANI) fibers were prepared by the combination of in-situ aniline oxidative polymerization and the hydrothermal methodology. The morphology of the prepared Ag/Co_3_O_4_@PANI ternary nanocomposite was studied by scanning electron microscopy and transmission electron microscopy, while the structural studies were carried out by X-ray diffraction and X-ray photoelectron spectroscopy. The morphological characterization revealed fibrous shaped PANI, coated with Ag and Co_3_O_4_ nanograins, while the structural studies revealed high purity, good crystallinity, and slight interactions among the constituents of the Ag/Co_3_O_4_@PANI ternary nanocomposite. The electrochemical performance studies revealed the enhanced performance of the Ag/Co_3_O_4_@PANI nanocomposite due to the synergistic/additional effect of Ag, Co_3_O_4_ and PANI compared to pure PANI and Co_3_O_4_@PANI. The addition of the Ag and Co_3_O_4_ provided an extended site for faradaic reactions leading to the high specific capacity. The Ag/Co_3_O_4_@PANI ternary nanocomposite exhibited an excellent specific capacity of 262.62 C g^−1^ at a scan rate of 3 mV s^−1^. The maximum energy and power density were found to be 14.01 Wh kg^−1^ and 165.00 W kg^−1^, respectively. The cyclic stability of supercapattery (Ag/Co_3_O_4_@PANI//activated carbon) consisting of a battery type electrode demonstrated a gradual increase in specific capacity with a continuous charge–discharge cycle until ~1000 cycles, then remained stable until 2500 cycles and later started decreasing, thereby showing the cyclic stability of 121.03% of its initial value after 3500 cycles.

## 1. Introduction

The depletion of fossil fuel resources, global warming, and the increasing demand of energy due to the rapid growth of the world’s population and industrialization have forced the scientific community to explore new alternative and green resources of energy [1,2]. The wind, tidal, and solar energies are alternative renewable energy resources that have attracted scientists to explore and utilize them because they are environmentally friendly and cost effective [3]. The major obstacle of these energy sources is the day–night and weather dependent performance. Therefore, some backup energy storage systems are required to be integrated with the renewable resources to obtain continuous energy supply. The fast pace in the growing trend of portable electronic devices and hybrid vehicles is another cause to explore efficient energy storage systems.

Presently, energy storage systems such as batteries and fuel cells are being widely used. The limitations of these systems are their low power delivery where a burst release of power is demanded [4]. Alternatively, the supercapattery has the ability to meet the high-power demand and also offers a long cyclic life. A supercapattery comprises of battery and capacitive type electrode materials, where charge is stored through two different mechanisms. In capacitive electrodes, charge is stored due to the formation of electrical double layers (EDL), whereas in battery electrodes, redox reactions are responsible for charge storage. Therefore, a supercapattery stores the energy through both mechanisms. The low energy density of a supercapattery can be improved by exploiting novel active materials in the development of supercapattery assembly [5,6].

Conducting polymers (CPs) have attracted much attention due to their peculiar properties, such as their pseudocapacitive features, facile synthesis protocol, good environmental and chemical stability, tunable conductivity, low production cost, etc. [7,8]. The major parameter for the evaluation of the performance of a device is its cyclic life, which depends upon the stability of the electrode materials during charge/discharge cycles [9]. CP as a standalone electrode material falls apart in this test due to the swelling and shrinkage phenomena, which induce breakage in the polymer backbone conjugated system, and hence restricts the charge propagation [10]. During the redox reaction, variations in the volume take place due to the insertion/de-insertion of dopants into/from the polymer. Hence, swelling, shrinkage and cracking of CPs during the process of charge propagation induce mechanical deterioration of the polymer structure causing the reduced electrochemical performance [11,12].

Therefore, to attain higher stability of the electrode material in terms of cyclic life, the nanoscale morphology plays an important role in altering the electrochemical properties. In the case of polyaniline (PANI), current density in voltammograms increases in the order nanospheres < nanorods < nanofibers. The morphology dependent effect versus scan rate has been reported [13]. Therefore, to address this issue, the development of tailor-made morphology of PANI and its novel ternary nanocomposite with electroactive metal oxide nanostructures can be a possible strategy.

A variety of transition metal oxides have been utilized as electrode materials in electrochemical applications due to their good electrochemical response and cyclic stability [14,15,16,17]. Cobalt oxide (Co_3_O_4_), among the transition metal oxides, has been widely used in many electrochemical applications due its abundance in nature, feasible synthesis, ease of tailor-made morphology, and favorable chemistry [18,19,20]. However, at nanoscale, the particles of Co_3_O_4_ aggregate, which leads to a reduction in the active sites, which ultimately results in the depletion of electroactivity of Co_3_O_4_ [21]. Therefore, the growth of these nanoparticles (NPs) on the polymer matrix can eliminate the issue of aggregation significantly.

On the other hand, a polymer nanocomposite with Co_3_O_4_ nanoparticles also enhances the conductivity and cycling stability of the nanocomposite [22]. Hence, the combination of Co_3_O_4_ and PANI results in a novel nanocomposite that may possess a good electrochemical signature as well as a better cyclic life. However, Co_3_O_4_ has a high band gap, which leads to poor conductivity and sluggish charge transfer. The issue can be resolved through the introduction of highly conductive elements, which can boost the electronic conductivity. Out of the noble metals, silver (Ag) has a good conductivity, thermal stability, and easy synthesis route to produce nanoparticles [23]. The decoration of polymer nanocomposites with Ag NPs enhances the electrochemical conductivity, thermal stability and optical and mechanical properties, resulting in a new class of polymer-based ternary nanocomposites suitable for electrochemical energy storage devices, especially for a supercapattery.

In this article, a facile synthesis of a novel ternary nanocomposite of Co_3_O_4_ nanograins and Ag nanoparticles decorated with fibrous polyaniline has been carried out. The prepared ternary nanocomposites and their counterparts, such as pure PANI and binary composite of Co_3_O_4_@PANI, were characterized by various analytical techniques such as X-ray diffraction (XRD), field emission scanning electron microscope (FESEM), energy dispersive spectroscopy (EDS), transmission electron microscope (TEM) and X-ray photoelectron spectroscopy (XPS). Electrochemical studies, such as cyclic voltammetry, galvanostatic charge–discharge and electrochemical impedance spectroscopy, were conducted in a standard three electrode cell. Finally, the performance evaluation of the fabricated device incorporating a battery-type ternary nanocomposite, Ag/Co_3_O_4_@PANI, as a negative electrode material and activated carbon as a positive electrode material was performed in a two electrodes assembly.

## 2. Experimental

### 2.1. Material

Aniline monomer was purchased from Sigma Aldrich (St. Louis, MO, USA). Analytical grade potassium permanganate (>99%), sulfuric acid (~98%), hydrochloric acid (~35%), phosphoric acid (~85%), ammonia solution (28%), and potassium persulfate (PPs) (~99%) were obtained from a local supplier (R & M Chemicals, Selangor Darul Ehsan, Malaysia). Cobalt chloride hexahydrate (CoCl_2_·6H_2_O), absolute ethanol (C_2_H_6_O ~99.8%), hydrogen peroxide (H_2_O_2_, 35%), poly (vinylidene fluoride) (PVdF), activated carbon (AC), and acetylene black were purchased from Sigma-Aldrich (>St. Louis, MO, USA). Silver nitrate (AgNO_3_, 99.9%) and potassium hydroxide (KOH) pallets were purchased locally. The anhydrous 1-methyl-2-pyrrolidinone (NMP) (99.5%) was obtained from Merck (Darmstadt, Germany). All analytical grade chemicals were consumed without any further treatment and solutions were prepared in de-ionized (DI) water.

### 2.2. Synthesis of Ag/Co_3_O_4_@PANI

PANI was synthesized by the oxidative polymerization of aniline in the presence of potassium persulfate (PPs) as an oxidizing agent. In a typical process, two solutions were prepared, firstly, 0.1 moles of aniline were dissolved in 250 mL of 1 M HCl and another solution containing 0.05 moles of PPs in 250 mL of 1 M HCl (aniline:PP ratio: 2:1). The solution of oxidant was slowly added into the solution of aniline, and the entire system was put on continuous stirring for 24 h, which resulted in greenish-black precipitates. The greenish-black precipitates of PANI was filtered and washed with excess DI water and ethanol to remove salts, oligomers, and other impurities. Then, PANI was de-doped with excess ammonia, thereafter, washed with excess water, ethanol and further doped with 1 M HCl to render it conductive. Thus, the obtained PANI emeraldine salt was dried at 50 °C in an oven for 12 h and stored in a desiccator for further use.

Pure Co_3_O_4_ was prepared by dissolving 1 millimole of CoCl_2_.6H_2_O in 35 mL DI water and into it 15 mL of ammonia solution (6%) was added very slowly dropwise while continuously stirring. The whole solution was stirred for an hour and then transferred to a Teflon lined hydrothermal rector. The hydrothermal cell was placed in a furnace at 150 °C for 5 h to obtain Co_3_O_4_ nanostructures. The synthesized Co_3_O_4_ nanograins were subjected to centrifugation to separate them from the solution and then washed with DI water and ethanol. Finally, they were dried at 100 °C for 12 h.

**Figure 1:**
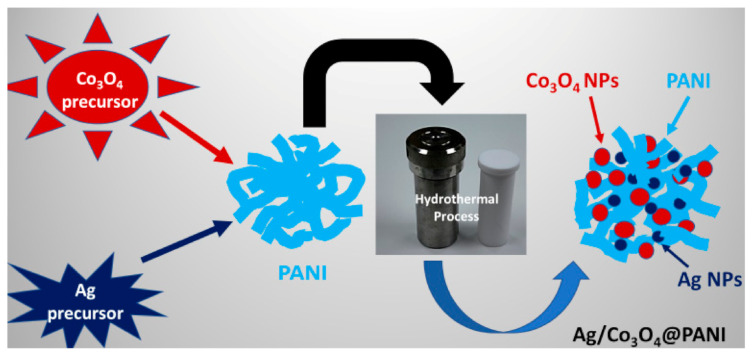
Schematic illustration of the synthesis of Ag/Co_3_O_4_@PANI ternary nanocomposite by the hydrothermal method.

To prepare the Co_3_O_4_@PANI binary nanocomposite, 1.0 g of already synthesized PANI was transferred to the above-mentioned mixture of CoCl_2_·6H_2_O and ammonia solution, and then the solution was poured into the hydrothermal cell and kept under similar conditions as discussed above to prepare Co_3_O_4_ nanograins. To prepare ternary nanocomposite, the mixture of CoCl_2_·6H_2_O, ammonia, PANI and AgNO_3_ (5 wt.% wrt CoCl_2_·H_2_O) was added to a hydrothermal cell after continuous stirring and afterwards subjected to hydrothermal reaction. Thus, the prepared Co_3_O_4_@PANI and Ag/Co_3_O_4_@PANI were washed with excess water and ethanol, de-doped and subsequently doped with 1 M HCl as in the case of PANI and finally dried at 100 *°C* for 12 h. Schematic illustration of the synthesis of Ag/Co_3_O_4_@PANI ternary nanocomposite by the hydrothermal method is shown in Figure 1.

### 2.3. Material Characterization

The surface morphological study and elemental scan were performed by FESEM (JEOL JSM-7600F, Tokyo, Japan) fitted with Oxford energy-dispersive X-ray spectroscopy (EDS, High Wycombe, UK). The size and shape of the ternary nanocomposite were examined by a transmission electron microscope (JEOL, ARM 7600, Tokyo, Japan). The phase identification and crystallinity of the prepared samples were conducted through a X-ray diffractometer (XRD) (Rigaku, Ultima IV, Tokyo, Japan) fitted with Cu-*Kα* X-ray radiation (λ = 1.5418 Å). The XRD spectra were scanned in the 2θ range of 10–80 degrees. X-ray photoelectron spectroscopy (XPS) (Versa Probe II, ULVAC-PHI, Inc. Chanhassen, MN, USA) studies were conducted under a ultra-high vacuum (~10^−10^ mbar) using monochromatic Al-*Kα* (*hν* = 1486.6 eV) and MultiPack software (version 9, Physical Electronics, Chanhassen, MN, USA) was used for the fitting of Gaussian–Lorentzian line shapes, estimation of chemical state and the spin-orbit splitting.

### 2.4. Electrode Fabrication and Electrochemical Measurements

The supercapattery devices were fabricated using the battery-type Ag/Co_3_O_4_@PANI ternary nanocomposite as a positive electrode and AC as a negative electrode. The electrodes were prepared by drop-casting the active materials on nickel foam with a covered area of 1 × 1 cm^2^. The positive electrode material slurry was prepared by blending Ag/Co_3_O_4_@PANI (75 wt.%), AC (15 wt.%), and PVdF (10 wt.%) in NMP and the mixture was stirred for 12 h under ambient conditions to achieve complete homogenization. The slurry was evenly distributed on nickel foam with the drop-casting technique, followed by drying at 90 °C for 12 h. The working electrodes for Co_3_O_4_@PANI and PANI were also prepared by the same method. The active material mass loading on all the prepared electrodes was ~5.00 ± 0.05 mg.

Cyclic voltammetry (CV) measurements were performed in the potential range 0–0.5 V at different scan rates (3–50 mV s^−1^), galvanostatic charge–discharge (GCD) at various current densities (1.5–4.5 A g^−1^) and electrochemical impedance spectroscopy (EIS) measurements at an AC voltage of 10 mV (*R.M.S.*, root mean square potential amplitude) and in the frequency range of 0.01 to 100 kHz using a potentiostat (Gamry Interface 1000 Instrument, Warminster, PA, USA) electrochemical work station in a three electrode cell system using Ag/AgCl as reference, platinum wire as counter, and coated nickel foam as working electrodes in 0.1 M KOH solution. For the performance comparison, Co_3_O_4_@PANI and PANI were also characterized systematically.

## 3. Results and Discussion

### 3.1. Morphological Characteristics

**Figure 2:**
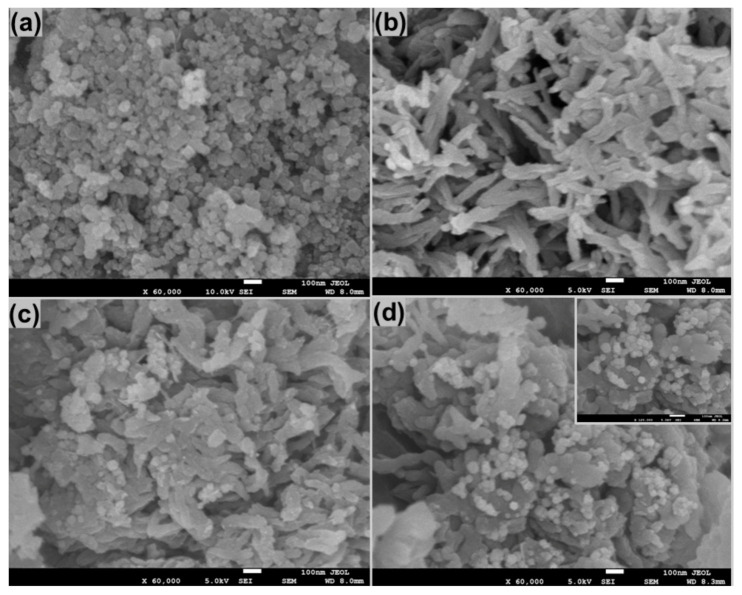
FESEM images of (**a**) cobalt oxide (Co_3_O_4_) nanoparticles (NPs), (**b**) polyaniline (PANI), (**c**), Co_3_O_4_@PANI binary nanocomposite (**d**), silver (Ag)/Co_3_O_4_@PANI ternary nanocomposite and inset of Figure 1d showing Co_3_O_4_ and Ag NPs decorated on PANI matrix.

**Figure 3:**
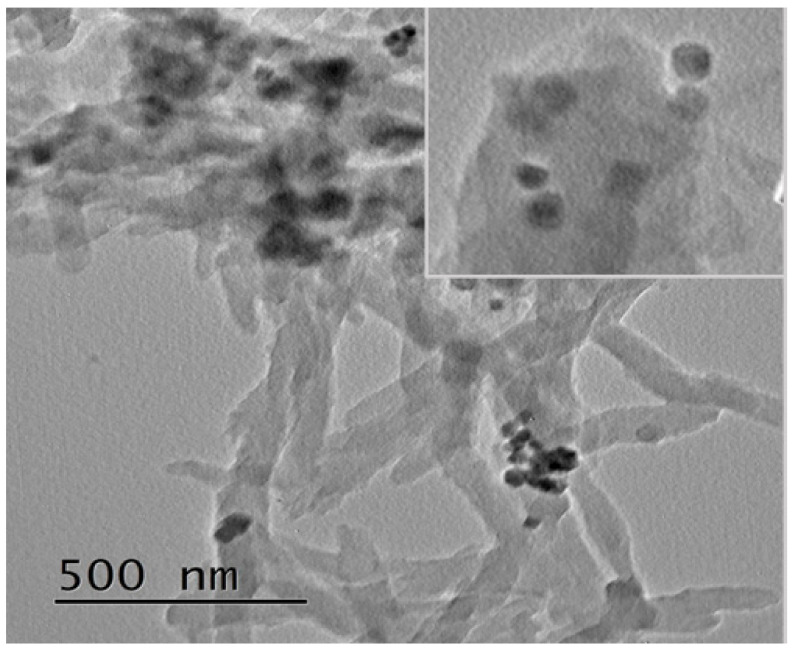
TEM image of Ag/Co_3_O_4_@PANI ternary nanocomposite and inset image focused on the Co_3_O_4_ and Ag nanoparticles embedded in the PANI matrix.

Figure 2 shows the FESEM images of Co_3_O_4_, PANI and Ag/Co_3_O_4_@PANI nanocomposites. Pure Co_3_O_4_ (Figure 2a) shows highly aggregated particles of different shapes, i.e., varying from spherical to cuboidal to hexagonal form and having intermittent morphologies. Apart from these, a few large sized lumps can be seen, which might be due to the stacking of one particle on the other or the growth of new particles on previously grown particles. On closer observation, it seems that the stacked particles are of the smallest dimensions amongst them all, which might be due to their much higher surface energy, thereby resulting in agglomeration. Another reason may be due to the incomplete/irregular crystal formation or seeds that failed to grow in a proper orientation, which result in an irregular or highly agglomerated structure [24]. PANI (Figure 2b) shows fibrous morphology, which might be due to its rapid mixing polymerization as explained by Ansari et al. [25]. The binary Co_3_O_4_@PANI shows Co_3_O_4_ particles sticking to PANI fibers as well as large number of Co_3_O_4_ particles, which can be seen in the vicinity or buried/trapped inside the interconnected PANI fibers. Similarly, in the ternary Ag/Co_3_O_4_@PANI nanocomposite, the Ag and Co_3_O_4_ nanoparticles are decorated on the PANI fibers or embedded inside the interconnected PANI fibers. It must also be mentioned that Ag, due to its very small size, is not distinctly visible and most of its particles are expected to be buried inside the matrix of PANI or Co_3_O_4_@PANI. The TEM image in Figure 3 also shows that PANI fibers are several micrometers in length with diameter <100 nm and the Co_3_O_4_ and Ag nanoparticles are well decorated as well as aggregated in different regions.

**Figure 4:**
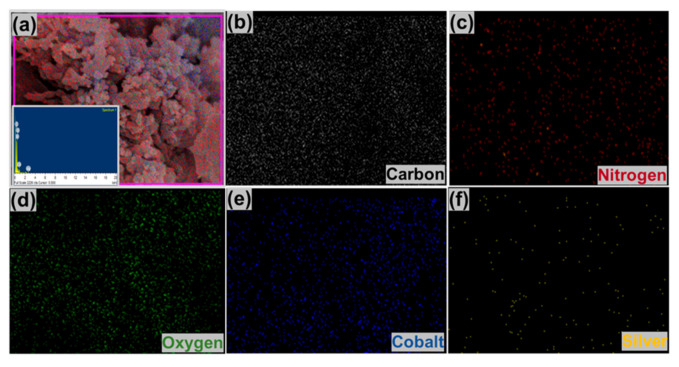
(**a**) EDS spectrum verifying the presence of the elements of the ternary nanocomposite (C, O, N, Co, and Ag) and showing scanned area image. EDS elemental mapping is presented by (b) C, (**c**) N, (**d**) O, (**e**) Co and (**f**) Ag contents of the Ag/Co_3_O_4_@PANI ternary nanocomposite.

The EDS (Figure 4) of Ag/Co_3_O_4_@PANI shows the presence of C, N, O, Co and Ag, while the elemental mapping shows the uniform distribution of the respective elements, thereby suggesting the efficacy of the synthesis methodology.

### 3.2. Structural Analysis

**Figure 5:**
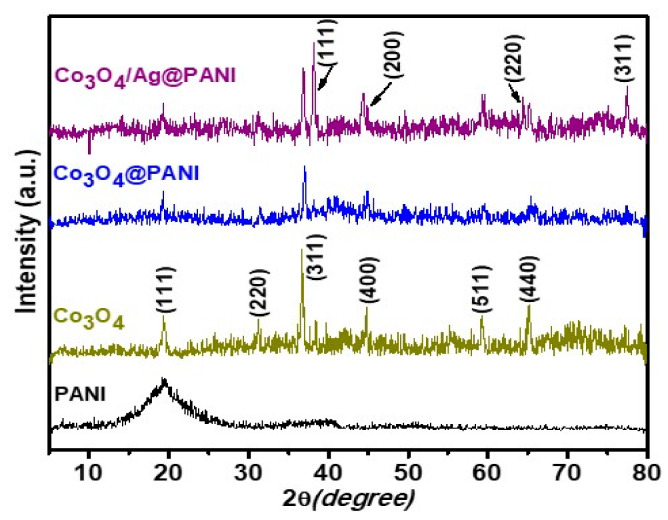
XRD pattern of PANI, Co_3_O_4_, Co_3_O_4_@PANI binary and Ag/Co_3_O_4_@PANI ternary nanocomposites scanned in the 2θ range of 5–80°.

The structural characteristics and crystallinity of PANI, Co_3_O_4_, Co_3_O_4_@PANI and the Ag/Co_3_O_4_@PANI nanocomposite was studied by XRD as depicted in Figure 5. The broad peaks in the XRD patterns of PANI show its highly amorphous nature with the observance of one distinct peak at 2θ value of 19.50° ascribed to a periodicity parallel to the polymer chain, which corresponds to the (010) plane [26]. Co_3_O_4_ demonstrated peaks at 2θ value of 19.38°, 31.32°, 36.94°, 44.65°, 59.25° and 65.00° due to the (111), (220), (311), (400), (511), and (440) planes, respectively, which confirmed the presence of cubic Co_3_O_4_ (JSCPD No. 42-1467) [27,28]. In the case of Co_3_O_4_@PANI, the above-mentioned peaks of Co_3_O_4_ were also present, while the peaks of PANI were not very distinct, which can be attributed to its low crystallinity as well as similar positioning near to the (111) peak of Co_3_O_4_. Similarly, in Ag/Co_3_O_4_@PANI, apart from PANI and Co_3_O_4_, additional peaks of Ag at 2θ value of 38.26°, 44.60°, 64.36° and 77.33° correspond to the (111), (200), (220), and (311) planes, respectively [28]. However, the peaks of Co_3_O_4_ and Ag after their incorporation in PANI were found with much reduced intensity and, originally, peaks of lower intensity were not distinctly visible in the nanocomposite, which might be due to the amorphous PANI that influences the degree of crystallization as mentioned in other reports [29].

### 3.3. XPS Analysis of Ag/Co_3_O_4_@PANI Ternary Nanocomposite

**Figure 6:**
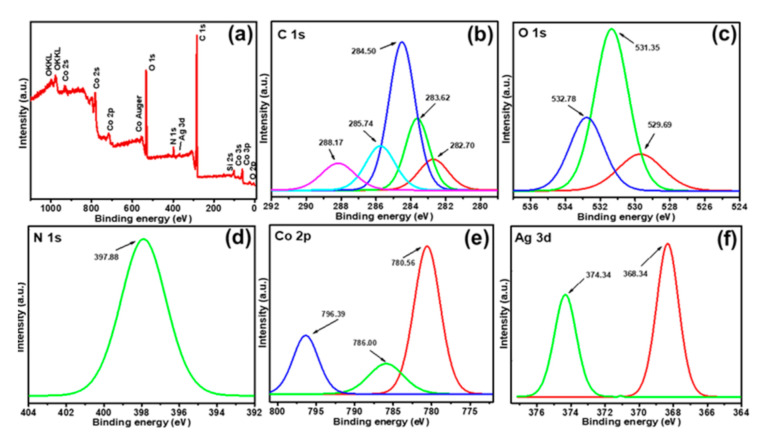
(**a**) XPS survey scan spectra of Ag/Co_3_O_4_@PANI. The representation of high-resolution spectra of C *1s* (**b**), O *1s* (**c**), N *1s* (**d**), Co *2p_3/2_* (**e**) and (**f**) Ag *3d*.

The surface composition and chemical states of the Ag/Co_3_O_4_@PANI nanocomposite was studied by XPS. The survey scan revealed the presence of C *1s*, O *1s*, N *1s*, Co *2p_3/2_* and Ag *3d_5/2_* without impurities (Figure 6). The carbon peak is due to the residual carbon from the sample and the instrument. The C *1s* spectra can be deconvoluted into five peaks at 282.70, 283.62, 284.50, 285.74 and 288.17 eV corresponding to C–H, C=C, C–C, C–O/C–NH and O–C=O, respectively, and can be attributed to the ring carbon, hydrogen and other functional groups of PANI. It might be interpreted that the functionalities, such as π electrons, C–O/C–NH and O–C=O in PANI, are the sites for interaction with Ag or Co_3_O_4_. The O *1s* spectra can be deconvoluted into three peaks at 529.69, 531.35 and 532.78 *eV* corresponding to the Co–O, hydroxide and structural water, respectively [30]. The broad N*1s* spectrum from 394.00 to 402.00 eV corresponds to the quinonoid, benzenoid, protonated benzenoid, and protonated quinonoid of PANI, thereby suggesting that PANI is conductive [31]. The Co *2p* spectrum consists of peaks at 780.00 and 795.00 eV corresponding to the Co^2+^ and octahedral Co^3+^. The weak satellite peak at 786.00 eV (i.e*.,* between 2*p_3/2_* and 2*p_l/2_* transitions) indicates that Co(II) and Co(III) co-exist in the sample, which also confirms the presence of Co_3_O_4_ in the sample [32]. Ag *3d* shows two deconvoluted peaks at 368.34 and 374.34 eV, corresponding to Ag *3d_5/2_* and Ag *3d_3/2_*, respectively. The difference of 6.00 eV between the binding energies is attributed to the characteristics of Ag, which also confirms the successful reduction of its AgNO_3_ salt to Ag under hydrothermal conditions [33].

### 3.4. Electrochemical Studies

#### 3.4.1. Cyclic Voltammetry Studies of PANI, Co_3_O_4_@PANI, Ag/Co_3_O_4_@PANI

**Figure 7:**
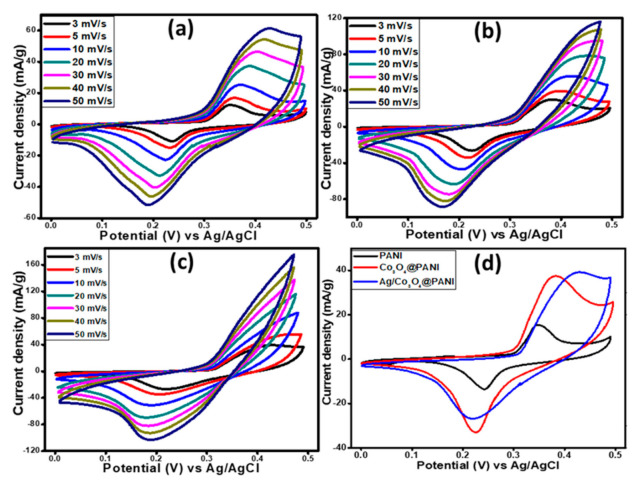
Cyclic voltammograms (CVs) of (**a**) PANI, (**b**) Co_3_O_4_@PANI binary, and (**c**) Ag/Co_3_O_4_@PANI ternary nanocomposites recorded at different scan rates, and (**d**) comparison CVs of PANI, Co_3_O_4_@PANI binary, and Ag/Co_3_O_4_@PANI ternary nanocomposites recorded at a low scan rate (3 mV s^−1^).

The cyclic voltammetry (CV) was carried out to analyze the voltametric character of the prepared Ag/Co_3_O_4_@PANI ternary nanocomposites in a typical three electrode system at room temperature and applied voltages ranging from 0 to 0.5 V. The material was scanned at 3, 5, 10, 20, 30, 40 and 50 mV s^−1^. Firstly, CV was performed for PANI and the obtained voltammogram is shown in Figure 7a with a well demarcated set of oxidation and reduction peaks that describe the direct relation of the current density with the scan rate. The direct relationship of current density and the scan rate is a clear explanation of the standard electrochemical charging and discharging property of the PANI [34,35,36]. A slow diffusion of ions to PANI could be evidenced by a little shift of cathodic and anodic extrema at each increased value of sweep rate. The ions can enter the internal structure of the PANI at a slower sweep but as the sweep rate was increased, the ions could only interact with the outer pores, which led to the shift in CV peak [37]. The maximum current density reached a value of 60 mA g^−1^ at maximum sweep rate of 50 mV s^−1^. Figure 7b displays the CV curves of the binary nanocomposite with a prominent increase in the current density from 60 to 120 mA g^−1^ at sweep rate of 50 mV s^−1^. The obtained voltammogram for the Co_3_O_4_/PANI binary nanocomposite, with clearly separated oxidation and reduction peaks, confirms its battery grade character with evidence of a reversible faradaic reaction that is distinct from the capacitive material [38,39,40]. The increased current density is obliged to the Co_3_O_4_ component in the binary nanocomposite and can be attributed to the enhanced area under the I-curve of Co_3_O_4_/PANI rather than the pure PANI, which supports the increased capacity because of the combined influence of Co_3_O_4_ with PANI [41]. The Co_3_O_4_ nanograins and conducting PANI form a smooth conductive path for the transportation of charge carriers along both Co_3_O_4_ and PANI by the hopping or tunneling mechanism, and hence an improved faradaic behavior has been observed [42]. The gradual increase in current densities was observed at a higher scan rate but the CV character remained unchanged, which is a clear evidence of battery grade behavior suitable for serving as an active material for energy storage applications [43]. The third CV studies were performed on the most envisaged Ag/Co_3_O_4_@PANI ternary nanocomposite and a remarkably high current density was obtained, in comparison to pure PANI and Co_3_O_4_@PANI binary nanocomposites (Figure 7c). The CV curve showed similar characteristics in PANI, binary, and ternary nanocomposites without any distortion in its cathodic and anodic peaks pattern, thereby further strengthening the battery grade behavior with faradaic reversibility. The high thermal and high electrical conductivity of Ag and Co_3_O_4_, in combination with PANI, clearly enhanced the current density of Ag/Co_3_O_4_@PANI due to the synergistic and additional effect. The highest value of achieved current density for ternary nanocomposites was credited to the introduction of Ag nanoparticles in the ternary nanocomposite. Figure 7d demonstrates the comparison at scan rate of 3 mV s^−1^ to distinctly analyze the increasing trend of current density in all three electrode materials i.e., PANI, Co_3_O_4_@PANI and Ag/Co_3_O_4_@PANI. The specific capacity, *Qs*, for all three samples was evaluated through the following expression in Equation (1) [20]:

(1) $$Q_{S}=\frac{1}{m\upsilon }\int_{\mathrm{Vi}}^{\mathrm{Vf}}I\;\times \;\mathrm{VdV}$$

where, *m* stands for the mass loading of the used electrode material measured in grams, *υ* indicates the sweep rate in V s^−1^, and the integral, with the limits from the initial to the final values of the potential, was taken over the area under the redox peak of the obtained voltammograms. The *Q_s_* values at a scan rate of 3 mV s^−1^ were obtained, which are 77.97 C g^−1^ for PANI, 207.00 C g^−1^ for Co_3_O_4_@PANI, and 262.62 C g^−1^ for Ag/Co_3_O_4_@PANI.

#### 3.4.2. Galvanostatic Charge–Discharge Studies (GCD)

**Figure 8:**
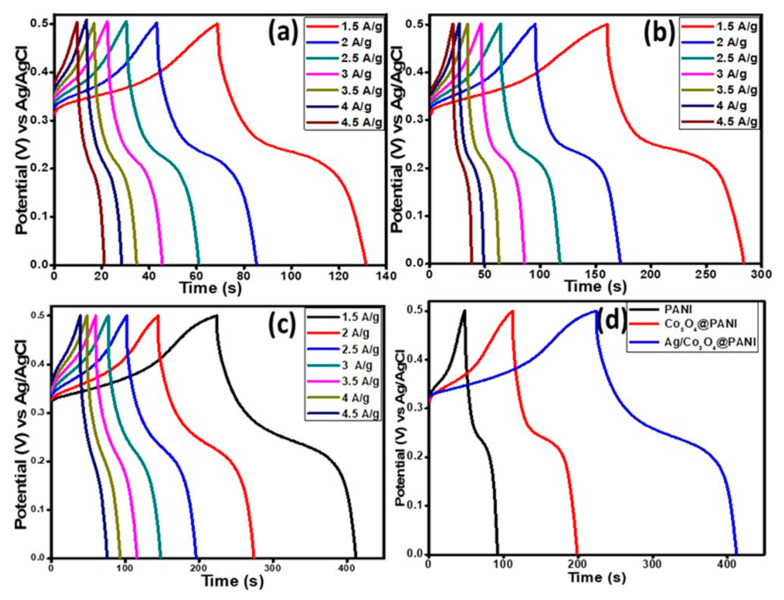
Galvanostatic charge–discharge (GCD) graph at various current densities, (**a**) PANI, (**b**) Co_3_O_4_/@PANI, (**c**) Ag/Co_3_O_4_@PANI, and (**d**) comparison of GCD curves for all three variants at a current density of 1.5 A g^−1^.

The GCD behavior was studied at various current densities for all three electrode materials (PANI, Co_3_O_4_@PANI, and Ag/Co_3_O_4_@PANI) and results are shown in Figure 8a–c. Their comparison is provided at a current density of 1.5 A g^−1^ as presented in Figure 8d. The ternary nanocomposite, Ag/Co_3_O_4_@PANI, demonstrated the best electrochemical performance in terms of longer discharge duration than the other two samples (PANI, Co_3_O_4_@PANI) due to the synergistic effect of Ag NPs along with the Co_3_O_4_ nanograin in the polymeric matrix. The values of specific capacity, *Q_s_* were also obtained through the GCD curves for all prepared three samples by using the following Equation (2) [44]:

(2) $$Qs=\frac{I\times \Delta t}{m}$$

where, ∆*t* stands for the discharge time interval in seconds and *m* indicates the mass loading of the prepared samples in grams and *I* denotes the current measurement in amperes. The calculated *Q_s_* values at a current density of 1.5 A g^−1^ are 95.76, 187.40 and 289.34 C g^−1^ and at a current density of 4.5 A g^−1^ are 54.50, 87.12 and 179.15 C g^−1^ for PANI, Co_3_O_4_@PANI, and Ag/Co_3_O_4_@PANI, respectively. From the *Q_s_* values obtained at the lowest and the highest current densities for all prepared samples, it can be interpreted that the ternary nanocomposite is the best performing battery-type electrode material for electrochemical energy storage systems due to the synergistic effect of the Ag and Co_3_O_4_ nanoparticles decorated on the fibrous PANI.

**Figure 9:**
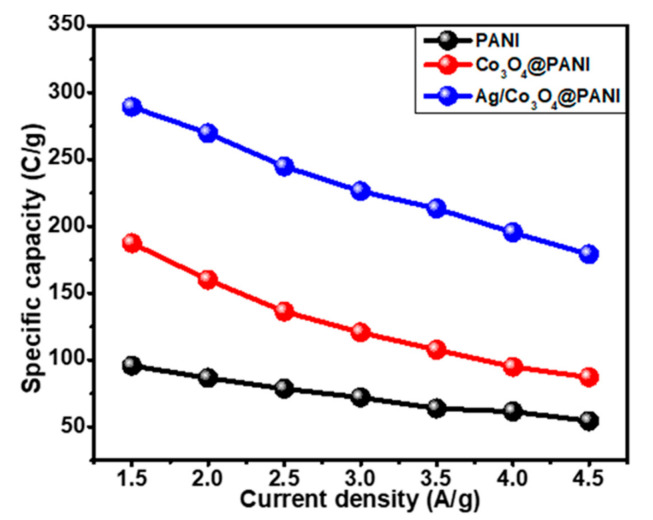
Specific capacity versus current density plot of PANI, Co_3_O_4_@PANI, and Ag/Co_3_O_4_@PANI nanocomposites.

The obtained specific capacities were plotted against the current densities and the graph obtained is shown in Figure 9 for all three samples. The trends in each of the three cases are similar and the nanocomposites showed higher specific capacities at lower current densities, which decreased gradually with the increase in current density. This behavior points to the fact that the OH^-^ ions transited partially on the prepared electrode. In contrast to PANI, an enhanced value of specific capacity is observed in the case of binary and ternary nanocomposites. This can be attributed to the highly dispersed or non-agglomerated conducting Co_3_O_4_ due to its growth on fibrous PANI, which resulted in the increased electroactive sites and facilitated the ion transportation through the prepared polymer matrix. The decoration of Ag further added a robust synergistic influence in the prepared Ag/Co_3_O_4_@PANI nanocomposite, thereby making it the best among all three active materials with an outstanding electrochemical performance.

#### 3.4.3. Electrochemical Impedance Spectroscopy Studies

**Figure 10:**
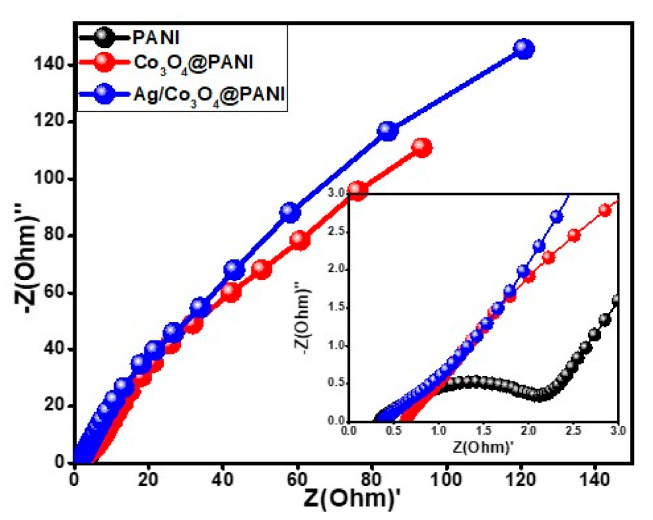
Nyquist plot of pure PANI, Co_3_O_4_@PANI, and Ag/Co_3_O_4_@PANI nanocomposites. Inset figure shows the enlarged high frequency region.

Figure 10 shows the Nyquist plots to testify the phenomena that occurred on the electrode surface in each of the three materials at an AC voltage of 10 mV (R.M.S.) and frequency range of 0.01 to 100 kHz. The plots of all three active materials showed straight lines at low frequencies and semicircles at high frequencies. The internal resistance of an active material is determined through the straight line at the point where it intersects the real axis in the region of high frequency, which depends upon three parameters (a) natural properties of the electrode materials, (b) the existed resistance at the point of contact of electrode and the collector material and (c) the ionic resistance offered by the electrolyte. The faradaic behavior is determined from the obtained semicircles, which depend upon the resistance in the transformation of the charges at the interface of electrode and the electrolyte [45]. The obtained straight line at low frequencies and the low electrochemical series resistance (ESR) value can be attributed to the incorporation of Co_3_O_4_ nanograin in PANI, which enhanced the electrochemical properties of the binary nanocomposite. The performed EIS study confirms the high performance of Ag/Co_3_O_4_@PANI due to the combined effect of Ag and Co_3_O_4_ nanostructures incorporated in the PANI matrix. The inset in Figure 10 depicts that the ternary nanocomposite has the smallest radius among all other electrode materials at low frequencies with a more vertical line at high frequencies parallel to the imaginary axis. This confirms the performance of Ag/Co_3_O_4_@PANI possessing the highest capacity of charge storage and least resistance against the charge transfer. The enhanced performance of Ag/Co_3_O_4_@PANI in contrast to PANI and Co_3_O_4_@PANI is due to the intercalation of conductive Ag and Co_3_O_4_ nanograins in PANI, which provides high permeability of electrolyte ions and eventually results in improved kinetics of charge transfer. The parameters such as equivalence (ESR) and charge transfer resistance have been summarized in Table 1. According to equivalent electrical circuit (E.E.C.) parameters, Ag/Co_3_O_4_@PANI demonstrated excellent performance in terms of equivalent series resistance (R_eq_) and charge transfer resistance (R_ct_).

## 4. Electrochemical Performance of Assembled Ag/Co_3_O_4_@PANI//AC Supercapattery

**Figure 11:**
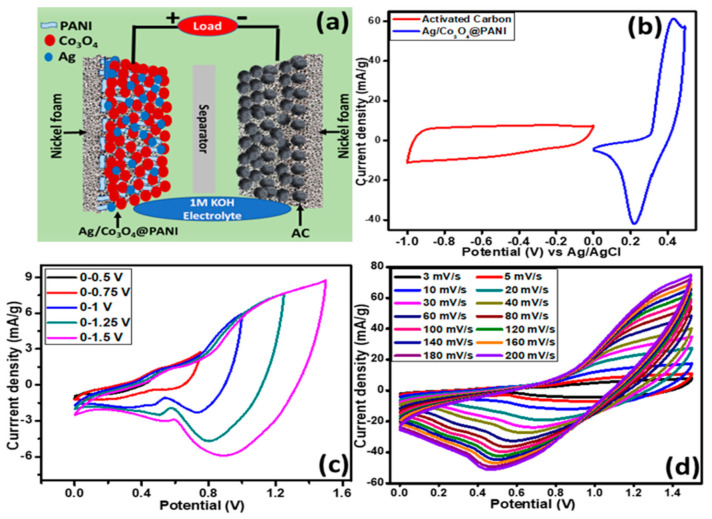
(**a**) Graphical design of the assembled supercapattery incorporating battery-type ternary nanocomposite, Ag/Co_3_O_4_@PANI, as a positive electrode, (**b**) CVs of activated carbon (AC) electrode at 5 mV s^−1^ and Ag/Co_3_O_4_@PANI electrode at scan rate of 3 mV s^−1^ in a three electrode cell system in 1 M potassium hydroxide (KOH), (**c**) CV curves of assembled Ag/Co_3_O_4_@PANI//AC device performed at diverse potential windows in 0.1 M KOH, and (**d**) CV curves of Ag/Co_3_O_4_@PANI//AC supercapattery at various scan rates from 3 to 200 mV s^−1^.

Two electrodes are usually assembled (negative and positive electrodes composed of capacitive and battery grade material, respectively) to construct a supercapattery (a compounded influence of a supercapacitor and a battery in a single device), to benefit from two different systems in a single device. A supercapattery was devised by taking Ag/Co_3_O_4_@PANI as a positive electrode and AC as a negative electrode as shown in Figure 11a. The higher potential window limits of the active materials (Ag/Co_3_O_4_@PANI) and activated carbon (AC) were determined independently by a three-electrode system. The CVs for each of the active and AC materials were run to assure the electrochemical (EC) signature individually, which is presented in Figure 11b. The potential windows (−1.0–0 and 0–0.5 V) for AC and the battery grade material are also shown in Figure 11b. The possible stable potential window obtained was 1.5 V through the combination of AC and battery grade material (Ag/Co_3_O_4_@PANI). The fabricated supercapattery (Ag/Co_3_O_4_@PANI//AC) in which Ag/Co_3_O_4_@PANI served as a positive electrode material and AC as a negative electrode material was then tested for EC studies in 0.1 M KOH electrolyte at room temperature. The CV scans were performed in various potential windows from 0 to 0.5 V to a maximum of 0 to 1.5 V in five sets of experiments to confirm the stable working potential of the assembled device as shown in Figure 11c. The performed CV studies are shown in Figure 11d at scan rates between 3 and 200 mV s^−1^ in a potential window of 0–1.5 V. The obtained voltammograms show no distortion in the typical anodic and cathodic peak patterns, instead showing enhanced current density as the sweep rates were increased, which proves that it follows the EC characteristics.

**Figure 12:**
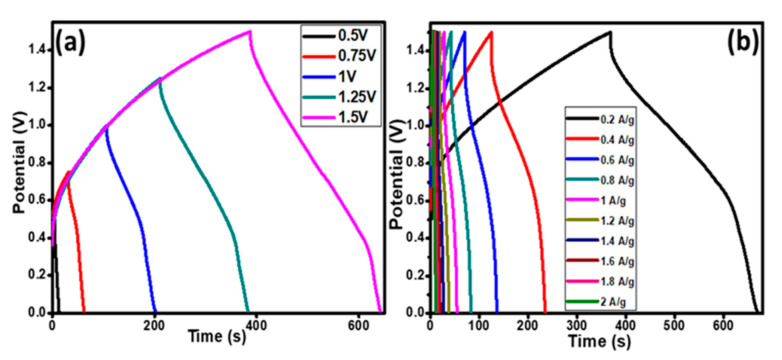
(**a**) Charge–discharge plots of the assembled device, Ag/Co_3_O_4_@PANI//AC supercapattery at different potentials at a current density of 0.2 A g^−1^ and (**b**) charge–discharge plots at different current densities ranging from 0.2 to 2.0 A g^−1^ at potential of 1.5 V.

The GCD studies were performed at various potentials from 0.5 to 1.5 V for the fabricated supercapattery at a current density of 0.2 A g^−1^ as shown in Figure 12a. The charge–discharge studies were carried out at a set of different current densities from 0.2 to 2.0 A g^−1^ as shown in Figure 12b. The obtained curves were nonlinear in behavior, which clearly points out the existence of the faradaic character of the device in the charge and discharge mechanism.

**Figure 13:**
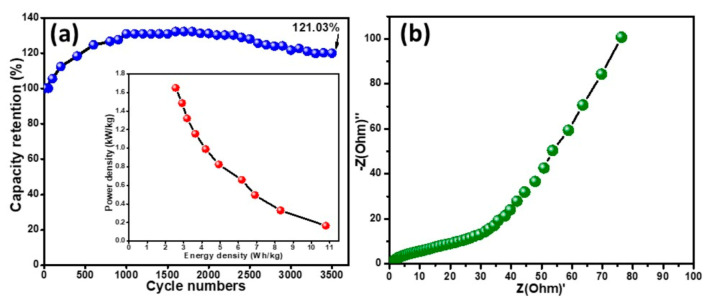
(**a**) The cycling stability studies of Ag/Co_3_O_4_@PANI//AC supercapattery. Inset in Figure 13(**a**) shows the energy density versus power density studies. (**b**) Nyquist plot of the assembled device.

The Ragone plot is presented to demonstrate the trend of power density (*P*) and energy density (*E*) as indicated in Figure 13a. The *E* and *P* were evaluated using Equations (3) and (4), respectively [46].

(3) $$E\left(\mathrm{Wh}/\mathrm{kg}\right)=\frac{\Delta V\times Qs}{2\times 3.6}$$

(4) $$P\left(W/\mathrm{kg}\right)=\frac{E\times 3600}{\Delta t}$$

where, Qs measured in C g^−1^ stands for the specific capacity, ΔV measured in volts symbolizes the applied potential, and Δt measured in seconds shows the total time taken for complete discharge.

The maximum energy and power density at a current density of 0.2 A g^−1^ was 14.01 Wh kg^−1^ and 165.00 W kg^−1^, respectively. The energy density trend was found to be inversely proportional to the power density and a decrease in current density was observed from 14.01 to 3.06 Wh kg^−1^ with the corresponding power density increase from 165.00 to 1650.00 W kg^−1^ as shown in the inset in Figure 13a. These parametric values assured the superior performance of the supercapattery assembled with the ternary nanocomposite, Ag/Co_3_O_4_@PANI, as an efficient electrode material. We compared our results with the reported literature on polyaniline composite electrodes, which is presented in Table 2.

Due to the swelling and shrinkage of conducting polymers, their viability as electrode materials has been questioned as they have encountered some issues that prevented their utilization in EC energy storage applications because during the charge and discharge mechanism their conductivity is depleted by the changes that occur in the backbone of the polymer chain. Therefore, the stability performance of the assembled device was monitored by running over 3500 charge–discharge cycles in 0.1 M KOH electrolyte as shown in Figure 13a.

The life cycle studies (Figure 13a) show that the specific capacity of the assembled device increases gradually up to 1000 charge–discharge cycles in the beginning, which may be due to the activation of the polymer composite resulting in the enhancement in the perforation of counter ions within the micropores of the active material [47]. A complete activation of the polymeric ternary nanocomposite was attained after 1000 cycles, after that there was stability in the trend, which might be due to the strong intercalation of the dopants in the polymer matrix, that helped to maintain the capacity retention. Therefore, there is no noticeable decay in the capacity retention from 1000 to 2500 GCD cycles and finally 121.03% capacity retention was observed after 3500 cycles. These results show the high stability of the PANI-based ternary nanocomposite, which makes it a suitable candidate for usage in the fabrication of high-performance energy storage devices.

Figure 13b shows the Nyquist plot and provides information on the charge transfer resistance and energy storage behavior of the assembled device. The above results and cyclic stability provide the foreground as a promising electrode material for supercapattery.

## 5. Conclusions

In summary, the Ag/Co_3_O_4_@PANI nanocomposites were prepared by the combination of in-situ oxidative polymerization and hydrothermal reaction. The FESEM images show sticking or embedding of Ag and Co_3_O_4_ nanoparticles to the PANI fibers. The XRD and XPS analyses of Ag/Co_3_O_4_@PANI showed peaks corresponding to Ag, Co_3_O_4_, and PANI, thereby suggesting the efficacy of the synthesis methodology. The CV was carried out to analyze the voltametric character through a typical three electrode system and applied voltages ranging from 0 to 0.5 V. The Ag/Co_3_O_4_@PANI battery type ternary nanocomposite showed the highest current density and specific capacity of 262.62 C·g^−1^ at 3 mV s^−1^ in comparison to 77.97 C·g^−1^ for PANI and 207.00 C·g^−1^ for Co_3_O_4_@PANI. The galvanostatic charge–discharge studies showed that the Ag/Co_3_O_4_@PANI ternary nanocomposite demonstrated the best electrochemical performance in terms of longer discharge duration. The Nyquist plots for the performed EIS study showed that Ag/Co_3_O_4_@PANI has the smallest radius among all other electrode materials at low frequencies with a more vertical line at high frequencies, parallel to the imaginary axis. This confirms that the Ag/Co_3_O_4_@PANI battery type material possesses the highest capacity of charge storage and least resistance against the charge transfer. The enhanced performance of Ag/Co_3_O_4_@PANI can be attributed to the high thermal and electrical conductivity of Ag and Co_3_O_4_, which assist in a smooth conductive path for the transportation of charge carriers along Ag, Co_3_O_4_ and PANI. Apart from this, the assembled supercapattery consisting of battery type ternary nanocomposite as a positive electrode and activated carbon as a negative electrode delivered a high energy density of 14.01 Wh kg^−1^ at a high-power density of 165.00 W·kg^−1^. The cyclic stability of the fabricated device showed the Ag/Co_3_O_4_@PANI battery type ternary nanocomposite retained 121.03% capacitance even after 3500 cycles. The high specific capacitance combined with excellent cyclic stability projects Ag/Co_3_O_4_@PANI as being a promising battery type electrode material for a high performance supercapattery.

## Figures and Tables

**Table 1:** Equivalent series resistance and charge transfer resistance of PANI, Co_3_O_4_@PANI, and Ag/Co_3_O_4_@PANI ternary nanocomposite.

Electrode	ESR (Ω)	R_ct_ (Ω)
PANI	0.32	2.02
Co_3_O_4_@PANI	0.64	0.74
Ag/Co_3_O_4_@PANI	0.38	0.8

**Table 2:** Performance of PANI composite electrodes.

Electrode Materials	Electrolyte	Specific Capacity	Energy Density	Application	Ref.
CoFe_2_O_4_/reduced graphene oxide/polyaniline composite	1M KOH	9 mF m^−1^ at 1 mA	270 × 10^−8^ Wh cm^−1^)	Supercapacitor	[48]
Graphene/polyaniline nanosheets	6M KOH	261.4 F/g at 100 mA/g		Three electrode system for Supercapacitor	[49]
Acetylene black-manganese cobaltite- polyaniline composite		0.35 F/cm^2^ at 1 mA/cm^2^	18.203 Wh/kg	Supercapacitor	[50]
Cobalt hydroxide/polyaniline hybrid nanostructure	1M NaOH	868 F/g at 10 mV/s		Pseudocapacitive electrode material	[51]
Mn_3_O_4_/polyaniline composite	6M KOH	352 F/g at 0.5 A/g	33.8 Wh/kg	Asymmetric supercapacitor	[52]
Manganese dioxide-polyaniline composite	Polyvinyl alcohol/KOH gel	129.2 F/g at 0.5 A/g	22.3 Wh/kg	Asymmetric supercapacitor	[53]
NiCo_2_S_4_/polyaniline nanosheets	Polyvinyl alcohol/KOH gel	152.06 F/g at 1 A/g	54.06 Wh/kg	Supercapattery	[54]
Strontium oxide/graphene/polyaniline ternary composite	1M KOH	151.66 C/g	33.8 Wh/ kg	Supercapattery	[55]
Metal organic framework (MOF)/polyaniline composites	1M KOH	162.5 C/g at 0.4 A/g	23.2 Wh/kg at 1 A/g	Supercapattery	[47]
Co_3_O_4_/Ag/polyaniline ternary composites	1M KOH	262.62 C/g at 3 mV/s	14.01 Wh/kg at 0.2 A/g	Supercapattery	Our work
